# Successful Antiviral Triple Therapy in a Longstanding Refractory Hepatitis C Virus Infection with an Acute Kidney Injury

**DOI:** 10.1155/2014/308729

**Published:** 2014-08-14

**Authors:** David Callau Monje, Niko Braun, Joerg Latus, Kerstin Amann, Mark Dominik Alscher, Martin Kimmel

**Affiliations:** ^1^Department of Internal Medicine, Division of Nephrology, Robert-Bosch-Hospital, Auerbach Street 110, 70179 Stuttgart, Germany; ^2^Department of Pathology, Erlangen University Hospital, Krankenhaus Street 8-10, 91054 Erlangen, Germany

## Abstract

*Introduction.* The HCV infection is a common disease with many chronically infected patients worldwide. So far, the standard therapy of a chronic HCV infection consisted of interferon as single therapy or in combination with ribavirin. After approval of the two protease inhibitors, boceprevir and telaprevir, the standard therapy for patients with genotype 1 changed. In patients with acute kidney injury (AKI) these therapies are not approved and have so far not been evaluated in studies. *Case Report.* In April 2012, a 58-year-old female was admitted due to a cryoglobulin-positive chronic HCV infection which had been treated with interferon and ribavirin. Currently, the patient was admitted because of severe complications with an acute kidney injury. We treated our patient successfully with a boceprevir based triple therapy. *Conclusion.* Limited data suggests that a therapy with ribavirin in patients with AKI seems to be safe under close monitoring. Our patient was treated successfully with a protease inhibitor based triple therapy. Nevertheless, it is necessary to plan an interventional study to evaluate the exact risk-benefit profile of triple therapy regimens in patients with AKI and hepatitis C.

## 1. Introduction

HCV infection is a very common disease with about 170 million chronically infected patients worldwide. Once a chronic infection develops, it is associated with high morbidity and mortality due to hepatic and extrahepatic involvement. Extrahepatic manifestations are a common phenomenon and are present in approximately 40% of all patients with chronic HCV infection [1]. These extrahepatic symptoms often manifest in a dermatologic, autoimmune, renal, or hematologic manner. Proofs of cryoglobulins are one of the most common findings in chronic HCV infection. Approximately 50% of all patients with chronic HCV infection are positive for cryoglobulins and, in case of an essential cryoglobulinemia in more than 90% of all cases, a chronic HCV infection is detectable [2].

So far the standard therapy of a chronic HCV infection consisted of pegylated or regular interferon alfa as a single therapy or in combination with ribavirin. After approval of the two protease inhibitors, boceprevir and telaprevir, in 2011, the standard therapy for patients with a genotype 1 changed. Triple therapy protocols were developed by maintaining the standard therapy containing of interferon alfa and ribavirin, by adding one of the new protease inhibitors. With these triple therapies, a relevant increase in sustained virologic response (SVR) rates was observed; SVR is defined as a negative HCV-RNA 24 weeks after cessation of antiviral therapy. The SVR rates in therapy naive patients increased from 40% to 67-68% [3] and, in formerly treated patients, from 21% to 59–66%  [4].

In patients with chronic kidney disease (CKD) and a glomerular filtration rate (GFR) of less than 50 mL/min, these therapies are not approved due to a contraindication of ribavirin and have so far not been evaluated in studies. KDIGO (Kidney Disease: Improving Global Outcomes) recommends, in case of an HCV-associated glomerulopathy with a decreased GFR, a monotherapy with pegylated interferon alfa [5]. In the most recent German guidelines for chronic HCV infection there is a grade B recommendation for a therapy with standard or pegylated interferon alfa as a monotherapy or in combination with low-dose ribavirin with controls of the blood count in close intervals [6]. This recommendation concurs to the results of a multicenter study, where pegylated interferon was used successfully in combination with low-dose ribavirin in patients with Hepatitis C infection receiving hemodialysis [7]. In one single-center study, the triple therapies were safely used in patients with CKD [8]. So far, no data exists about the use of a triple therapy in patients with acute kidney injury and cryoglobulinemic vasculitis.

## 2. Case Report

In April 2012, a 58-year-old female was admitted to our hospital due to a cryoglobulin-positive chronic HCV infection. The chronic HCV infection (genotype 1b) was first diagnosed in July 2007 and was treated according to guidelines from September 2007 over 24 weeks with pegylated interferon alfa and ribavirin. In the course of the therapy, a considerable drop of HCV-RNA in the blood occurred, but the HCV-RNA never dropped below the detection threshold (partial nonresponse). The antiviral therapy was, therefore, stopped according to the guidelines after 24 weeks.

Currently, the patient was admitted because of a dramatic decline in the general state of health. She complained about shortness of breath, peripheral edema, and an increase in body weight.

The examination revealed edema at the lower and upper limbs as well as anasarca. Furthermore, vasculitic skin efflorescences were found at the lower legs.

Laboratory tests showed an increased erythrocyte sedimentation rate (65 mm/h, norm 1–30 mm/h) and the serum creatinine was increased to 1,8 mg/dL (norm 0,5–1,2 mg/dL) according to an estimated GFR (MDRD equation) of 30 mL/min. A nephrotic syndrome with a proteinuria of 6,9 g/24 h, a serum albumin of 2,2 g/dL (norm 3,5–5,0 g/dL), and elevated lipids (LDL 272 mg/dL, norm 100–150 mg/dL) was diagnosed. Liver function tests were within normal range, but HCV-RNA was positive with 5 million copies/mL. Complement factors C3 (75 mg/dL, norm 90–180 mg/dL) and C4 (2,7 mg/dL, norm 10,0–40,0 mg/dL) were decreased and cryoglobulins (type IgM) were positive. A liver ultrasound showed sonographic signs of a beginning liver cirrhosis.

**Figure 1:**
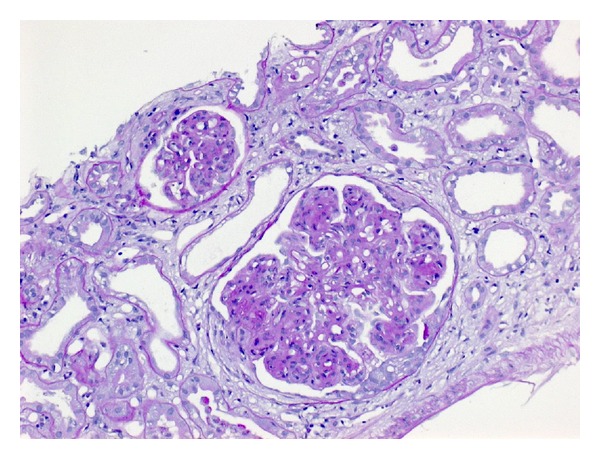
PAS stained renal biopsy of our patient showing the characteristic histologic features of a MPGN type 1 (thickening of the basement membrane, diffuse mesangial expansion, and proliferation). Furthermore, a discrete extracapillary proliferative component and a diffuse tubulointerstitial damage were present.

After recompensation with loop diuretics with a decrease in body weight of 9 kg, we performed a kidney biopsy. Histological workup showed a severe diffuse membranoproliferative glomerulonephritis (MPGN) type 1 with a discrete extracapillary proliferative component and a diffuse tubulointerstitial damage (Figure 1). The immunofluorescence study revealed granular deposits of IgG, IgM, and C3c alongside the glomerular basement membrane while IgA was negative. The final electron microscopy confirmed the diagnosis and showed a partially doubled glomerular basement membrane with interposition of mesangial cells, fusion of the podocyte foot processes, and intracapillary proliferation.

Because of a severe chronic HCV infection with hepatic and extrahepatic manifestations, we decided to treat our patient with a boceprevir based triple therapy. We initiated the therapy with a 4-week lead-in phase with pegylated interferon alfa 2a and ribavirin. Due to renal impairment, we decreased the interferon dose to 135 *μ*g weekly and the ribavirin dose to 200 mg daily. After control of the virologic response and decrease of the HCV-RNA to 6.000 IU/mL, we added boceprevir 800 mg 3 times daily. Due to the beginning liver cirrhosis, the triple therapy was continued for a total of 48 weeks.

**Figure 2:**
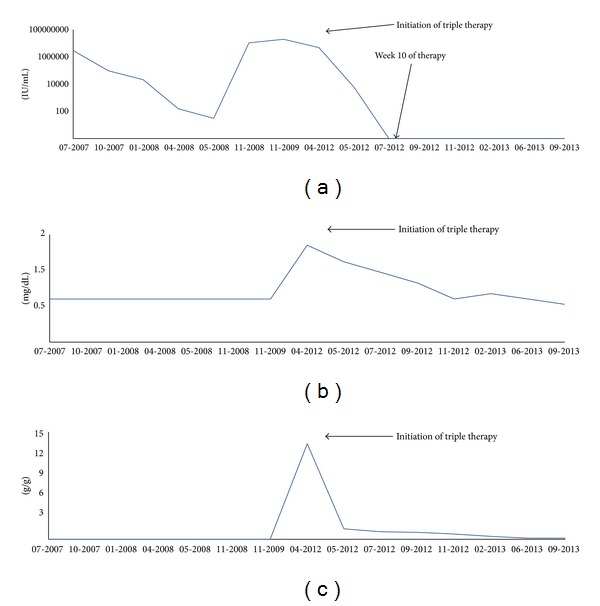
Trend of crucial laboratory parameters. (a) HCV-RNA blood levels in IU/mL. After initiation of triple therapy, the levels fell continuously and were negative for the first time after 10 weeks of therapy. (b) Serum creatinine blood levels in mg/dL. At admission, the creatinine was considerably elevated and fell constantly after start of therapy. (c) Proteinuria in g protein/g creatinine. Quickly after initiation of antiviral therapy, proteinuria decreased dramatically and remained low over the whole time of surveillance.

During the antiviral therapy, the administration of erythropoetin was necessary due to anemia. After 10 weeks of therapy duration, the viral load and the cryoglobulins were negative for the first time. The creatinine level dropped within the normal range and the proteinuria declined from initially 12.5 g protein/g creatinine to 0.4 g protein/g creatinine (Figure 2).

The triple therapy was discontinued after a total therapy duration of 48 weeks. HCV-RNA remained negative during the whole surveillance and a SVR was declared 24 weeks after cessation of therapy.

## 3. Discussion

Our patient received an appropriate antiviral therapy with a partial nonresponse in the past. Currently, we diagnosed a chronic HCV infection with severe hepatic and extrahepatic manifestations with acute kidney injury. In general, KDIGO suggested that all patients with renal impairment and HCV infection should be evaluated for antiviral therapy [9]. The decision whether to treat or not should be based on the potential benefits and risks of therapy. Because of the severity of the hepatic and extrahepatic manifestations in our patient and a partial nonresponse to interferon alfa and ribavirin, we chose a new triple therapy regimen adding boceprevir to pegylated interferon alfa and ribavirin. With this decision, we followed an expert recommendation about retherapy in patients with partial virologic response in patients without renal impairment [10].

Concerning the weekly doses of pegylated interferon alfa, we followed the German HCV guidelines which recommend 90–135 *μ*g weekly [6] which is in line with a recommendation in a recently published review about Hepatitis C therapy in patients with renal impairment [11]. Most of the studies which investigated the use of pegylated interferon alfa in patients with impaired kidney function used 135 *μ*g weekly [12]. However, there was only one randomized study with 85 patients which compared 90 *μ*g to 135 *μ*g weekly and detected so far no differences in SVR [13]. Given that most studies investigated 135 *μ*g weekly, we chose 135 *μ*g pegylated interferon alfa as weekly dose.

In terms of ribavirin, the guidelines recommend an initial dose of 200 mg every other day with increase of doses to a maximum of 200–400 mg daily in patients with renal impairment [6]. We chose 200 mg as starting dose with respect to studies in dialysis patients [14].

Our patient developed anemia without a hemolytic component; therefore, an erythropoetin therapy was initiated.

## 4. Conclusion

In summary, growing data suggests that a therapy with ribavirin in patients with renal impairment seems to be safe under close monitoring. Our patient with severe renal and extrarenal manifestations was treated successfully (normalization of kidney function, dropped proteinuria, and restitution of vasculitic skin findings) with a protease inhibitor based triple therapy and still has an SVR 30 weeks after cessation of antiviral therapy.

Nevertheless, it is necessary to plan an interventional study to evaluate the exact risk-benefit profile of a triple therapy regimen in patients with acute kidney injury and hepatitis C. Till then the triple therapies could be administered to such patients only on an individual case decision.
